# Recent Progress in Artificial Intelligence in Biosensor Development: From Bioprobe Design to Fabrication and Signal Analysis

**DOI:** 10.3390/bios16070382

**Published:** 2026-07-13

**Authors:** Yunseon Han, Haebin Jo, Minyoung Ju, Seowoo Bae, Ju Young Kim, Jinho Yoon, Taek Lee

**Affiliations:** 1 Department of Chemical Engineering, Kwangwoon University, 20 Gwangwoon-Ro, Nowon-Gu, Seoul 01897, Republic of Korea; hys02100@kw.ac.kr (Y.H.); haebin7945@kw.ac.kr (H.J.); minyoung9585@kw.ac.kr (M.J.); suwoo0602@kw.ac.kr (S.B.); kjy05155@kw.ac.kr (J.Y.K.); 2 Department of Biomedical-Chemical Engineering, The Catholic University of Korea, Bucheon-si 14662, Republic of Korea

**Keywords:** artificial intelligence, biosensors, bioprobe engineering, sensor fabrication, signal interpretation

## Abstract

The coronavirus disease 2019 (COVID-19) pandemic highlighted the need for rapid, accurate, and point-of-care diagnostic technologies, accelerating interest in biosensors as next-generation analytical platforms. However, biosensor performance is governed by a connected sequence of processes, including bioprobe–target recognition, sensor fabrication, structural optimization, and signal interpretation. Because these processes involve multiple interacting variables, conventional empirical approaches often have limitations in efficiently optimizing biosensor performance and interpreting complex analytical signals. Artificial intelligence (AI) and machine learning (ML) provide tools to model these relationships and support prediction-guided biosensor development. This review discusses recent progress in AI-assisted biosensor development in three sequential stages. First, AI-assisted bioprobe design is reviewed, including in silico aptamer discovery, smart-SELEX-based aptamer screening, and peptide receptor design for improving molecular recognition. Second, AI-driven sensor fabrication and structural optimization are discussed, focusing on electrochemical feature extraction, paper-based microfluidic device optimization, and optical biosensor parameter prediction. Third, ML-based signal analysis is examined as a strategy for converting complex electrochemical, colorimetric, and optical responses into quantitative analytical outputs. By organizing these examples as a connected workflow rather than as separate applications, this review highlights how AI can link molecular design, device engineering, and signal interpretation to accelerate the development of next-generation biosensors.

## 1. Introduction

Biosensors are platforms that combine biological recognition elements with signal transducers to analyze the presence and properties of target analytes [1,2,3,4,5]. Selective interactions between the target analyte and the bioprobe convert biomolecular-level information into measurable signals, enabling not only the identification of target presence but also qualitative and quantitative analysis of its concentration and state [6,7,8]. Biosensors have been applied in diverse fields, including clinical diagnostics, environmental monitoring, food safety, and infectious disease detection, and have become useful analytical platforms for rapid analysis and point-of-care testing [9,10,11,12]. Conventional laboratory-based analytical methods, including chromatography, immunoassays, molecular diagnostics, and spectroscopy-based techniques, provide high accuracy and reproducibility, yet they rely heavily on sample pretreatment, multistep reactions, expensive instrumentation, and skilled personnel [13,14,15,16]. These limitations have increased the need for alternative platforms that offer simpler analytical procedures, rapid responses, miniaturization potential, and suitability for point-of-care use, and biosensors have consequently attracted attention as promising alternatives that can meet these demands [17,18,19]. This need becomes even greater when information that changes continuously or in real time must be captured rapidly in complex biological environments, making biosensor platforms capable of immediate signal transduction and analysis increasingly important [20,21].

Recent biosensor research has moved beyond simple target detection and is increasingly focused on the more precise measurement and interpretation of complex signal changes associated with target binding [22,23,24]. Electrochemical sensors employ measurement techniques such as electrochemical impedance spectroscopy (EIS), cyclic voltammetry (CV), and differential pulse voltammetry [25,26]. Optical sensors also employ surface plasmon resonance (SPR)-based analysis [27,28]. These techniques improve detection sensitivity and analytical precision by more sensitively capturing subtle signal changes induced by interfacial reactions or molecular binding [29,30]. In real complex biological matrices, non-specific binding, signal noise, and cross-reactivity can act simultaneously and cause signal distortion [31,32]. Under multivariable conditions, such complex behavior is difficult to explain sufficiently using only simple threshold-based discrimination or linear models [33,34]. This is because biosensor responses do not arise from a single binding event alone, but rather from the sequential coupling of interfacial reactions, charge and mass transport, and signal amplification after molecular recognition [35,36]. Binding affinity and binding kinetics are important for target recognition, and the state of surface immobilization and the reaction environment also determine interfacial accessibility and reaction efficiency. At the same time, material composition, nanomaterial structure, and electrode surface state alter the active area and charge-transfer pathways, thereby affecting the magnitude and stability of the final response [37,38,39]. Biosensor performance should therefore be understood as a complex outcome arising from the combined effects of multiple design variables and their interactions, and biosensor design needs to be treated inherently as a high-dimensional optimization problem [40,41]. From this perspective, empirical trial-and-error approaches have clear limitations in efficiency and reproducibility for systematically exploring the design space.

Recent advances in artificial intelligence (AI) and machine learning (ML) have established these approaches as key tools for addressing high-dimensional nonlinear problems [42,43,44]. AI can learn relationships among variables and predict optimal conditions within a design space, and its effectiveness has been demonstrated consistently in design and analytical problems that require multivariable optimization and interpretation of complex signals [45,46]. In protein and molecular design, sequence–structure–function relationships are now being learned to support both binding affinity prediction and candidate generation [47,48]. In bioprocessing and general process optimization, interactions among multiple variables and performance outcomes are being modeled in ways that reduce dependence on repeated experiments while improving productivity and process stability [49,50]. These trends suggest that biosensor development can also benefit from more systematic and predictive approaches to bioprobe design, fabrication optimization, and the interpretation of complex sensor signals [51,52,53].

From this perspective, this review systematically examines how AI and ML are being integrated into biosensor design and fabrication. In biosensors, AI-based approaches can extend beyond improving the efficiency of individual steps and support the integration of the entire development process [54,55]. Biosensor performance is determined by multiple design and fabrication variables and their interactions, so AI can be used to explore combinations of molecular recognition elements and materials at the design stage, learn relationships between process variables and sensor responses at the fabrication stage to derive optimal conditions, and connect complex signals to quantitative diagnostic information at the interpretation stage [56,57]. These advances in AI are exerting an increasingly important influence on biosensor development and provide a basis for moving biosensors beyond simple signal detection devices toward intelligent analytical systems in which design, fabrication, and interpretation are integrated [58,59]. Unlike previous reviews that mainly discuss AI applications in biosensor signal processing, classification, or individual sensor platforms, this review frames AI-assisted biosensor development as a connected workflow spanning bioprobe design, sensor fabrication and structural optimization, and signal analysis. In this workflow, AI is not treated as an isolated computational tool applied after sensor fabrication, but as a design framework that links molecular recognition, device engineering, and analytical interpretation. This perspective allows us to evaluate how AI contributes to each stage of biosensor development and how information from one stage can guide decisions in the next.

## 2. AI-Driven Optimization of Biosensor Design

The detection performance of biosensors largely depends on their ability to selectively recognize target molecules, which is closely associated with the binding properties of bioprobes. Molecular recognition elements, such as antibodies, aptamers, and peptides, have long been used as key components of biosensors. Their development, however, has traditionally relied on iterative experimental screening strategies, including immunoassays [60], systematic evolution of ligands by exponential enrichment (SELEX) [61], and phage display [62]. Although these approaches can generate effective recognition candidates, they are often time- and cost-intensive because large candidate libraries must be screened [63]. Their efficiency may also vary depending on the structural complexity and biochemical characteristics of the target molecule [64]. As biosensor technologies become increasingly sophisticated, bioprobe development is required to move beyond the identification of binding candidates and to simultaneously consider affinity, specificity, structural stability, and compatibility with sensor platforms [65,66].

Against this background, AI and in silico modeling have emerged as important tools for shifting bioprobe development from empirically driven screening toward prediction-guided design (Table 1). Data-driven approaches can integrate sequence, structural, and physicochemical information to estimate the likelihood of target–bioprobe interactions before experimental validation, thereby enabling more refined narrowing of candidate pools at the pre-experimental stage [67]. This transition is significant not merely because it accelerates molecular search processes, but because it reorganizes the design rationale of molecular recognition elements more quantitatively and systematically. AI-driven bioprobe engineering should therefore be regarded not simply as a supporting technology for the early stages of biosensor development, but as a central framework that reshapes the design of molecular recognition elements governing overall sensor performance. From this perspective, this section focuses on how AI is being incorporated into the discovery and design of bioprobes.

### 2.1. In Silico Aptamer Design and Sensor Integration: AI-Assisted Aptamer Generation and Binding Prediction

**Figure 1:**
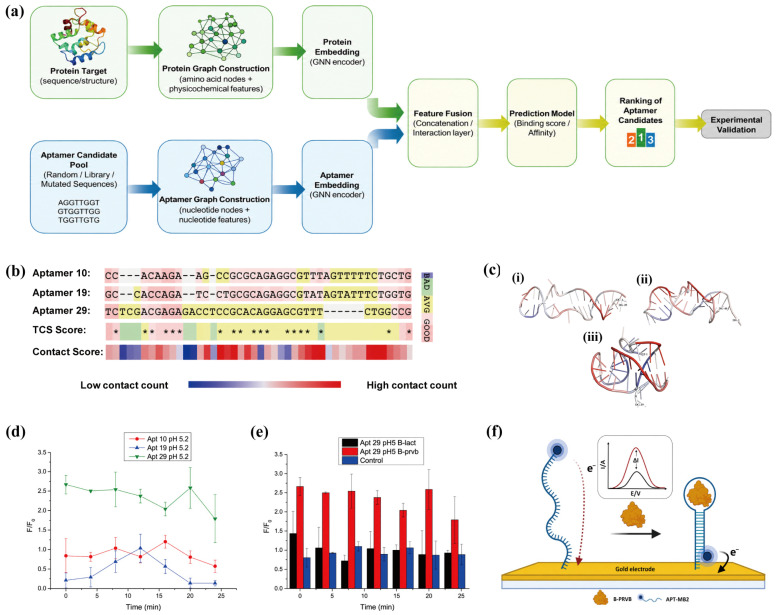
AI-assisted aptamer discovery and biosensor implementation for β-parvalbumin (β-PRVB) detection. (**a**) In silico aptamer selection workflow. (**b**) Sequence alignment and nucleotide-level contact score analysis of selected aptamer candidates. Asterisks (*) indicate conserved nucleotide positions. (**c**) Predicted lowest-energy three-dimensional (3D) structures of three selected aptamers: (i) Aptamer 10, (ii) Aptamer 19, and (iii) Aptamer 29. (**d**) Time-dependent fluorescence response of APT29 to β-PRVB at pH 5.2. (**e**) Selectivity evaluation of APT29 using β-PRVB, β-lactoglobulin (β-Lact), and control. (**f**) Electrochemical detection scheme of β-PRVB using the APT29-based aptasensor. Adapted with permission from Ref. [68]. Copyright 2026, Abril et al.

Abril et al. proposed an in silico aptamer platform for detecting β-parvalbumin (β-PRVB), a major fish allergen [68]. They generated a total of 6213 aptamer candidates using a random mutation algorithm and a variational autoencoder and employed a graph neural network to model aptamers and proteins as molecular graphs and predict the apparent dissociation constants between the two molecules. In this context, AI-based candidate generation and binding prediction can be used as a strategy to reduce the experimental screening burden associated with aptamer development [51,74]. The candidate pool was subsequently filtered based on the predicted dissociation constant, folding free energy, and secondary structure, from which three aptamers with high structural stability and binding potential were selected as primary candidates (Figure 1a). These three candidates were further compared using sequence alignment, nucleotide-level contact scores, and lowest-energy three-dimensional structures (Figure 1b,c). The tertiary structures of the aptamers generated using 3dDNA and the β-PRVB structure predicted by AlphaFold2 were then used for HADDOCK docking analysis to examine the structural interactions between the protein and aptamers. Structure-based docking analysis can be used to predict the binding modes between aptamers and proteins [75], and the actual binding capability was subsequently validated using a fluorescence quenching assay. Among the candidates, APT29 showed the highest fluorescence recovery at pH 5.2 and a selective response toward β-PRVB, leading to its selection as the final aptamer (Figure 1d,e). APT29 was then immobilized on a gold electrode and implemented as a square wave voltammetry (SWV)-based electrochemical aptasensor (Figure 1f), achieving a limit of detection (LOD) of 3.3 μg/mL and a limit of quantification (LOQ) of 10 μg/mL, which demonstrated its applicability to commercial processed food samples. Overall, the role of AI in this study was not limited to computationally expanding the search space for candidate aptamer sequences but extended to evaluating their binding potential and structural suitability before experimental validation. This work is significant because it improved the efficiency of bioprobe development by enabling the systematic design of aptamers applicable to electrochemical biosensors through an AI-driven in silico pipeline, rather than relying solely on complex SELEX-based iterative screening.

### 2.2. Selectivity-Oriented Aptamer Screening and Optimization: AI-Assisted Interaction Prediction and Cross-Reactivity Assessment

**Figure 2:**
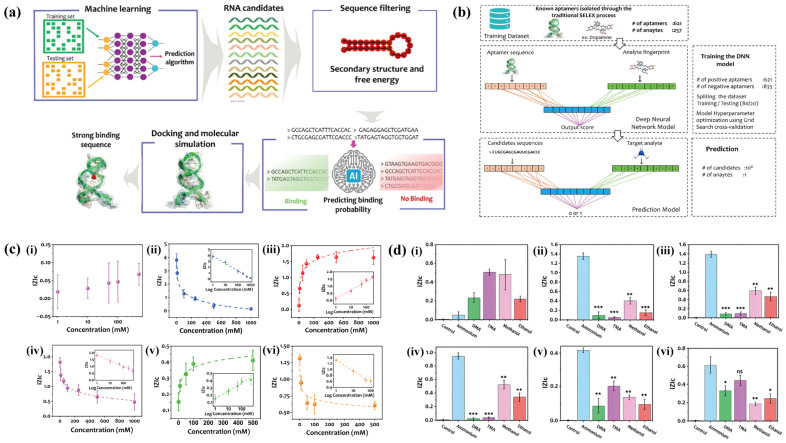
AI-assisted smart systematic evolution of ligands by exponential enrichment (smart-SELEX) design and validation of RNA aptamers for NH_4_^+^ detection. (**a**) Overall smart-SELEX workflow integrating machine learning (ML)-based binding prediction, structural filtering, docking, and final aptamer selection. (**b**) Convolutional neural network (CNN)-based binding prediction model, where aptamer sequences and molecular fingerprints are processed through separated CNN blocks to estimate binding probability. The symbol “#” denotes the number. (**c**) EIS-based sensor responses versus log NH_4_^+^ concentrations for the control sequence and the five smart-SELEX-selected aptamers: (i) control, (ii) aptamer 1, (iii) aptamer 2, (iv) aptamer 3, (v) aptamer 4, and (vi) aptamer 5. (**d**) Selectivity evaluation of the top-ranked aptamers against interfering molecules (dimethylamine, trimethylamine, methanol, ethanol): (i) control, (ii) aptamer 1, (iii) aptamer 2, (iv) aptamer 3, (v) aptamer 4, and (vi) aptamer 5 (* *p* ≤ 0.5, ** *p* ≤ 0.05, *** *p* ≤ 0.0001, ns—not significant). Adapted with permission from Ref. [70]. Copyright 2022, Douaki et al.

Douaki et al. presented a Smart-SELEX-based strategy for designing RNA aptamers for ammonium ion detection [70]. Considering the Debye length of electrochemical sensors, they generated a random library consisting of 10^8^ RNA sequences with a length of 27 nucleotides, followed by primary filtering based on secondary structure and free energy. The binding potential toward ammonium ions was then evaluated using docking and molecular dynamics simulations, while possible cross-binding with dimethylamine (DMA) and trimethylamine (TMA) was also examined. On this basis, five RNA aptamer candidates were finally selected (Figure 2a). In parallel, previously reported aptamer data for small-molecule targets were collected to construct a training dataset comprising 1456 entries, in which aptamer sequences and target information were separately introduced into convolutional neural network blocks (Figure 2b). Such approaches that jointly learn aptamer sequence information and target molecular information can be used to predict aptamer–target interactions in a data-driven manner [74]. The five selected aptamers were subsequently applied as biorecognition elements in electrochemical aptasensors, and their concentration-dependent responses to ammonium ions were evaluated based on EIS-derived impedance changes. Some candidates exhibited distinct impedance changes in the low-concentration range, suggesting their potential as bioprobes for ammonium ion detection (Figure 2c). The sensor responses were also compared against interfering substances, including DMA, TMA, methanol, and ethanol. The final aptamer candidates showed relatively large impedance changes toward ammonium ions, whereas their responses to the other interfering substances were limited (Figure 2d). These results indicate that selectivity-oriented design should consider not only binding affinity toward the target, but also the possibility of cross-reactivity with structurally similar non-target molecules [65]. From this perspective, the study by Douaki et al. can be regarded as an example showing that AI-driven bioprobe engineering can extend beyond affinity-centered candidate discovery and incorporate selectivity required in practical sensor environments as an explicit design variable. However, the interfering substances considered as negative targets during the design stage were limited to DMA and TMA. Future studies should therefore include a broader range of interfering molecules at the design stage to further improve selectivity under complex real-sample conditions.

### 2.3. AI-Designed Peptide Probes and Data-Driven Signal Interpretation: AI-Assisted Peptide Screening and Voltammogram Analysis

**Figure 3:**
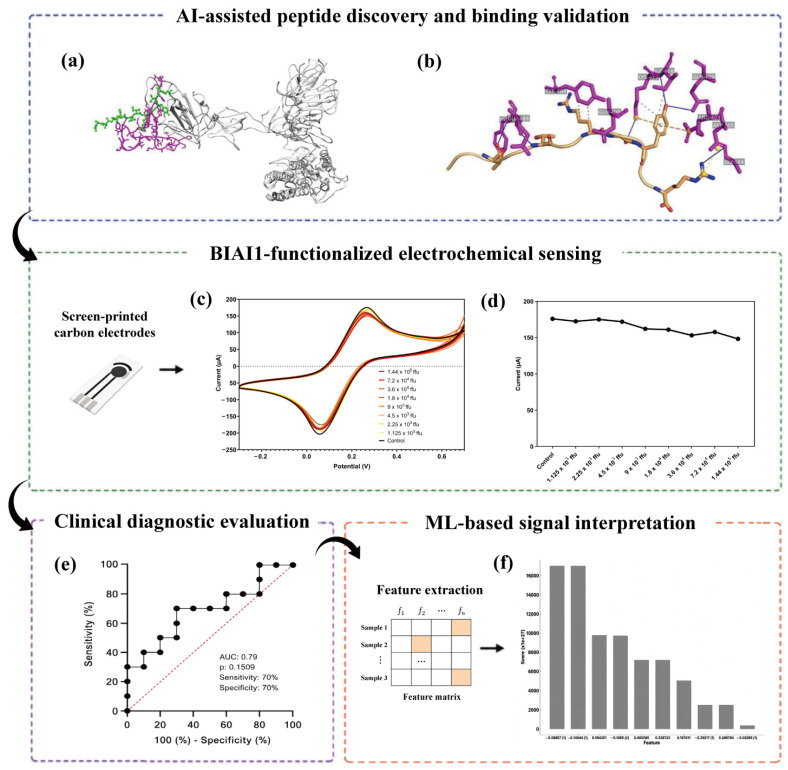
AI-designed peptide receptor and ML-assisted electrochemical biosensing for SARS-CoV-2 detection. (**a**) Predicted binding pose of BIAI1 with the SARS-CoV-2 Spike receptor-binding domain (RBD) obtained through molecular docking analysis. The Spike protein, BIAI1, and RBD are shown in grey, green, and pink, respectively. (**b**) Key molecular interactions between BIAI1 and the Spike-RBD complex identified through molecular docking analysis. Hydrogen bonds are indicated by dotted lines. (**c**) Representative voltammograms of the BIAI1-based sensor in response to serially diluted SARS-CoV-2 samples. (**d**) Signal response of the BIAI1-based sensor according to viral load. (**e**) ROC analysis showing the diagnostic performance of peak-current-based classification in clinical saliva samples. (**f**) SHAP analysis of the neural network model using raw data of the reduction curve. Adapted with permission from Ref. [69]. Copyright 2025, Garcia-Junior et al.

Garcia-Junior et al. reported a biosensor platform that combines AI-based peptide receptor design with ML-based electrochemical signal interpretation for the detection of severe acute respiratory syndrome coronavirus 2 (SARS-CoV-2) in saliva [69]. Peptide-based bioreceptors can be designed to recognize binding sites on protein targets [76,77]. In this study, this concept was applied to the receptor-binding domain (RBD) of the SARS-CoV-2 spike protein, and Bio-Inspired Artificial Intelligence Peptide 1 (BIAI1) was identified through candidate screening using the surrogate-assisted genetic algorithm for peptide evaluation and prediction (SAGAPEP) platform. HPEPDOCK-based molecular docking subsequently suggested that BIAI1 could form hydrogen-bonding and electrostatic interactions with key amino acid residues in the RBD (Figure 3a,b). The selected BIAI1 was functionalized on a rhodamine 6G-modified screen-printed carbon electrode and applied as the biorecognition element of an electrochemical biosensor. Changes in electrochemical signals according to viral concentration were analyzed using CV (Figure 3c,d). In serial dilution experiments, the sensor achieved a LOD of 1.61 × 10^4^ focus-forming units (ffu) and a LOQ of 4.89 × 10^4^ ffu. In clinical saliva samples, however, receiver operating characteristic (ROC) analysis based solely on peak current yielded an area under the curve (AUC) of 0.79, with sensitivity and specificity of approximately 70% (Figure 3e). This result suggests that a single peak-current descriptor may be insufficient to fully capture diagnostic information in complex saliva matrices [78]. To overcome this limitation, the authors further introduced ML-based signal interpretation as a complementary downstream strategy to enhance the diagnostic discrimination of the BIAI-functionalized biosensor in complex saliva samples. Using voltammogram data as input, they compared multiple ML algorithms and found that a neural network model trained on raw reduction-curve data achieved the best performance, with sensitivity, specificity, and accuracy all reaching 90%. Shapley additive explanations (SHAP) analysis further identified the potential region near the reduction peak as a major discriminative feature, suggesting that ML-based interpretation may exploit voltammogram patterns that are difficult to capture using a single electrochemical descriptor (Figure 3f). Overall, the study by Garcia-Junior et al. demonstrates that AI-driven bioprobe engineering can be extended to peptide receptor design, while the complementary incorporation of ML-based signal interpretation further improves diagnostic discrimination in complex biological samples.

Taken together, these examples indicate that AI is no longer limited to a supporting role in reducing the number of candidates during bioprobe discovery. Instead, its role is expanding toward integrating sequence generation, binding prediction, structural stability assessment, and selectivity evaluation within a unified design workflow. In aptamer design, large sequence spaces can be computationally explored, allowing promising candidates to be prioritized before experimental validation. Smart-SELEX-based approaches further enable more refined selectivity-oriented design by considering not only target-binding affinity, but also potential cross-reactivity with interfering molecules [79,80]. In peptide receptor design, AI-based candidate generation has also related to ML-based electrochemical signal interpretation, demonstrating that biorecognition element design and the improvement of sensor readout performance can be addressed as a continuous problem [81,82,83]. These developments suggest that bioprobe engineering can no longer be understood solely as a repetitive process of empirical screening followed by experimental validation. Rather, it is shifting toward a data-driven molecular design stage in which sequence, structure, binding properties, and sensor applicability are considered together [84,85,86]. Many current studies, however, remain based on limited training datasets and specific target classes; future research should therefore expand these approaches to broader molecular groups and more complex sample environments [87,88]. Nevertheless, these strategies are significant because they are transforming the design of molecular recognition elements, which critically determine biosensor performance, into a more predictable and reproducible process. Importantly, these AI-designed bioprobes do not represent an endpoint of biosensor development. Rather, they provide the molecular-recognition foundation for the subsequent optimization of sensor materials, device structures, fabrication conditions, and signal-transduction processes.

## 3. AI-Assisted Sensor Fabrication and Structural Optimization

After AI-assisted bioprobe design, the next stage of biosensor development is to translate molecular recognition into stable and measurable sensor responses through material selection, structural design, fabrication conditions, and signal interpretation. Biosensor performance is not determined solely by the binding affinity of molecular recognition elements. Actual sensor responses arise from the combined effects of material composition, surface structure, interfacial reactions, signal-transfer pathways, and operating conditions, and sensor fabrication and design therefore need to be understood as nonlinear optimization problems involving multiple interacting variables [89,90,91]. This complexity appears in different forms depending on the platform. In electrode-based electrochemical sensors, metal composition and surface formation govern charge transfer and analytical signals [92,93,94], whereas in paper-based microfluidic devices, channel structure and operating conditions determine signal intensity and readout accuracy [95,96]. In optical biosensors, structural parameters and optical properties define resonance behavior and sensitivity [97,98,99]. The dominant physical mechanisms differ across platforms, yet a common feature is that sensor performance is determined by multiple design variables and their interactions. AI and ML have recently emerged as key tools for quantitatively addressing these multivariable optimization problems [42,100,101]. Some studies have applied ML to extract important features from electrochemical signals and improve analytical performance on that basis [102,103], whereas others have used cyclic optimization of microfluidic paper-based analytical devices (μPAD) structure and operating conditions to reduce experimental trial and error [104,105,106]. In the optical biosensor field, approaches have also been proposed to accelerate design by using ML to rapidly predict key performance parameters that would otherwise require repeated numerical simulations [107]. These trends show that AI is no longer limited to simple post-processing, but is increasingly linking biosensor fabrication, device engineering, and signal interpretation into a more continuous and predictive workflow.

### 3.1. Dual-Metal Electrochemical Sensing: AI-Assisted Feature Extraction from Multipeak Signals

**Figure 4:**
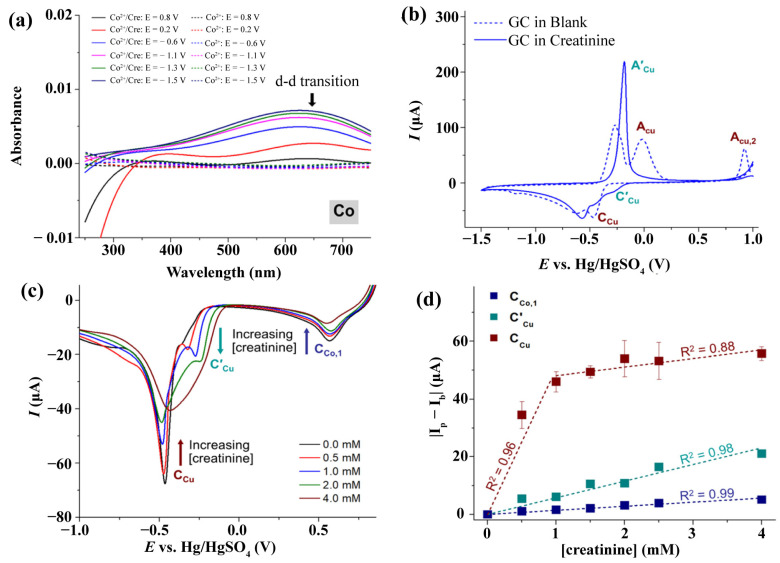
Data-driven extraction of electrochemical features from multi-peak voltammetric responses for ML-assisted creatinine sensing. (**a**) Spectroelectrochemical absorbance responses associated with Co–creatinine complex formation. (**b**) Cyclic voltammograms of the GC electrode in blank electrolyte and creatinine solution. (**c**) Concentration-dependent cyclic voltammograms obtained in creatinine solutions. (**d**) Blank-subtracted peak currents extracted from representative redox peaks and their use as ML input descriptors for Random Forest-based creatinine prediction. Adapted with permission from Ref. [92]. Copyright 2025, Kaewket et al.

In electrochemical sensors that rely on complex formation with metal ions, the metal layer formed on the electrode surface can be oxidized to metal ions at positive potentials, and the generated ions can induce characteristic redox responses by forming complexes with the analyte at the electrode–electrolyte interface [108,109]. Based on this principle, Kaewket et al. proposed a dual-metal electrochemical sensor for creatinine detection using a Co@Cu/GC electrode, in which copper nanoparticles were first electrodeposited onto a glassy carbon electrode, followed by the sequential deposition of cobalt nanoparticles [92]. Under sufficiently positive potentials, the surface Cu and Co species can be oxidized to Cu^2+^ and Co^2+^, respectively, and these ions generate electrochemical responses through complex formation with creatinine near the electrode surface [110]. The interaction between creatinine and each metal species has been supported by previous electrochemical and spectroelectrochemical observations. In the Cu^2+^/creatinine system, cyclic voltammetry shows that the copper-related reduction behavior changes in the presence of creatinine [111], while spectroelectrochemical analysis reveals absorbance changes associated with Cu–creatinine complex formation [112,113]. Similarly, in the Co^2+^/creatinine system, the cobalt-related voltammetric response changes in the presence of creatinine [111,114], and corresponding spectroelectrochemical results support Co–creatinine complex formation [92]. These observations suggest that Cu and Co do not interact with creatinine in an identical manner but instead provide complementary electrochemical responses over different potential ranges. This complementary behavior was reflected in the electrode-level response of the Co@Cu/GC sensor. Bare GC does not show a distinct redox peak for creatinine, whereas Cu/GC exhibits a copper–creatinine-related peak [115,116], and Co/GC shows changes in cobalt-related peaks [117,118]. In contrast, the Co@Cu/GC electrode shows both copper- and cobalt-associated responses in the presence of creatinine, with some signals enhanced compared with the corresponding single-metal electrodes (Figure 4a) [92]. This indicates that the two metals do not simply provide independent signals but can jointly modify the local electronic environment and interfacial reaction pathway, resulting in more informative analytical responses than a single-metal system [119]. The formation of these electrochemical features is also affected by the electrode fabrication conditions. In electrode-based electrochemical sensors, metal composition, surface formation, particle distribution, and the degree of active-site exposure are known to jointly influence charge transfer and analytical signals [120,121,122]. In the Co@Cu/GC system, therefore, the deposition condition is not merely a fabrication variable, but an important design factor that determines the intensity and distribution of the redox and complex-derived signals generated during sensing. Under the optimized conditions, the Co@Cu/GC electrode exhibited multiple cathodic peaks that changed systematically with increasing creatinine concentration (Figure 4b). To make the link between the raw voltammetric data and ML analysis clearer, representative cathodic peaks were annotated and used for feature extraction (Figure 4c). The blank-subtracted peak currents obtained from the selected peaks showed different concentration-dependent trends, indicating that each peak contained partially distinct analytical information (Figure 4d). In addition to individual peak heights, peak ratios were calculated to capture relative changes among the redox features.

These peak heights and peak ratios were then used as input descriptors for Random Forest regression [92]. This feature-based strategy differs from conventional single-peak calibration because the model can exploit complementary information distributed across multiple electrochemical responses. In electrochemical sensors, combinations of multiple features can reflect analyte concentration or chemical state more robustly than a single signal, and ML can serve as a useful tool for quantitatively interpreting such complex signal patterns [123,124]. Therefore, this example is not presented here to emphasize creatinine-specific chemistry in detail, but to illustrate how feature-rich electrochemical responses generated at engineered metal interfaces can be transformed into structured ML inputs for quantitative sensing. On this basis, the authors defined peak height and peak ratio as features and compared the predictive performance of individual features using Random Forest regression (Table 2).

The results showed that features including peak ratios could be more effective for predicting creatinine concentration than a single peak current alone. Ratio-based descriptors such as CCo,1/C′Cu, C′Cu/CCu, and C′Cu/ACu showed higher testing R^2^ and lower testing RMSE values than most individual peak-current features. This result can be interpreted by considering the electrochemical meaning of the ratio features. A single peak current mainly reflects the intensity of one redox process, whereas a peak ratio describes the relative change between two electrochemical responses generated at the dual-metal interface. Therefore, peak ratios can partially compensate for common signal variations, such as differences in electrode loading, active surface area, or overall current intensity, while preserving the concentration-dependent balance between copper- and cobalt-related responses. The stronger performance of the ratio features suggests that creatinine concentration is better represented by the relative distribution of multiple redox signals than by the absolute intensity of one peak alone. The authors then used this multifeature set as the input for a comparison of regression models, including linear regression (LR), support vector machine (SVM), k-nearest neighbors (kNN), decision tree (DT), random forest (RF), extra trees (ET), gradient boosting (GB), and extreme gradient boosting (XGB) [125,126], and confirmed that the complex voltammetric response could be used effectively for creatinine concentration prediction (Table 3).

Therefore, the significance of this study lies in integrating electrode design and data-driven analysis into a single sensing strategy by generating multiple electrochemical signals through dual-metal electrode design and converting them into a feature set that can be interpreted quantitatively through ML-based signal interpretation. This approach is meaningful in that it extends the analysis of electrochemical sensors beyond a single-peak-centered framework toward multifeature representations that can capture complementary information otherwise missed. Recent studies have likewise shown a growing use of strategies that extract features from electrochemical signals and interpret them with ML to improve analytical performance [127,128,129]. This case can therefore be understood as an example showing that electrode surface design and signal interpretation can be treated not as separate steps, but as closely connected problems.

### 3.2. Paper-Based Microfluidic Devices: AI-Driven Multivariable Optimization

In paper-based microfluidic analytical devices, design and operating variables such as channel width, sample volume, and reaction time directly determine signal intensity and readout accuracy [130,131]. Rather than addressing this problem through single-variable adjustment, Kangzheng Lv et al. adopted a cyclic optimization strategy that combines computer vision, a backpropagation neural network, and a genetic algorithm [95]. The proposed CNGCOS is structured as a closed-loop framework in which paper-based colorimetric signals are extracted through image analysis and then used to adjust the key parameters of the μPAD iteratively.

**Figure 5:**
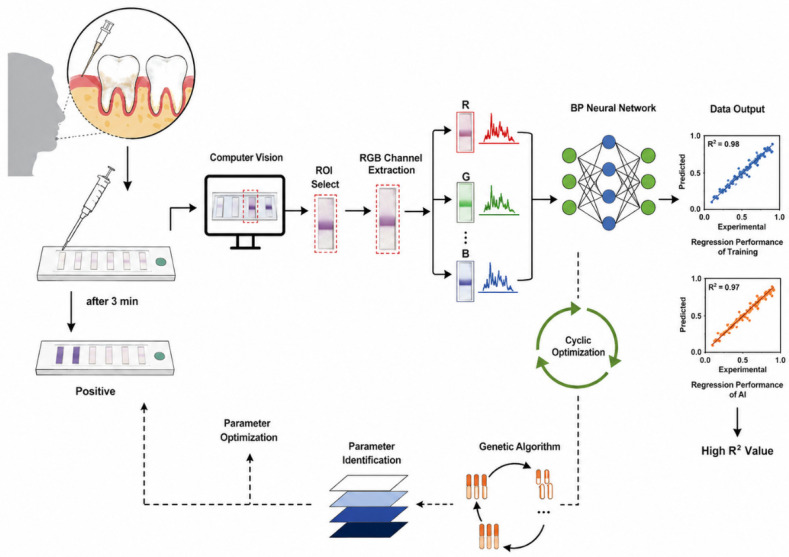
Schematic illustration for ML-driven CNGCOS to optimize μPADs. Adapted with permission from Ref. [95]. Copyright 2025, Kangzheng Lv et al.

The overall workflow first proceeds through sample introduction, color generation, image acquisition, region-of-interest selection, RGB channel extraction, BP neural network training, and genetic algorithm-based cyclic optimization, and is meaningful in that it attempts to shift μPAD fabrication from empirical trial and error toward a data-driven design problem (Figure 5) [132]. Unlike conventional single-factor optimization, in which one variable is changed while the others are fixed, this cyclic optimization strategy treats Hb concentration, test strip width, sample volume, and reaction time as coupled variables. This is important because sequential single-factor testing can require repeated experimental rounds and may overlook optimal conditions when the effect of one parameter depends on the values of the others. The authors defined hemoglobin (Hb) concentration, test strip width, sample volume, and reaction time as input variables, and set color intensity (CI) and colorimetric distance (CD) as the optimization targets.

**Figure 6:**
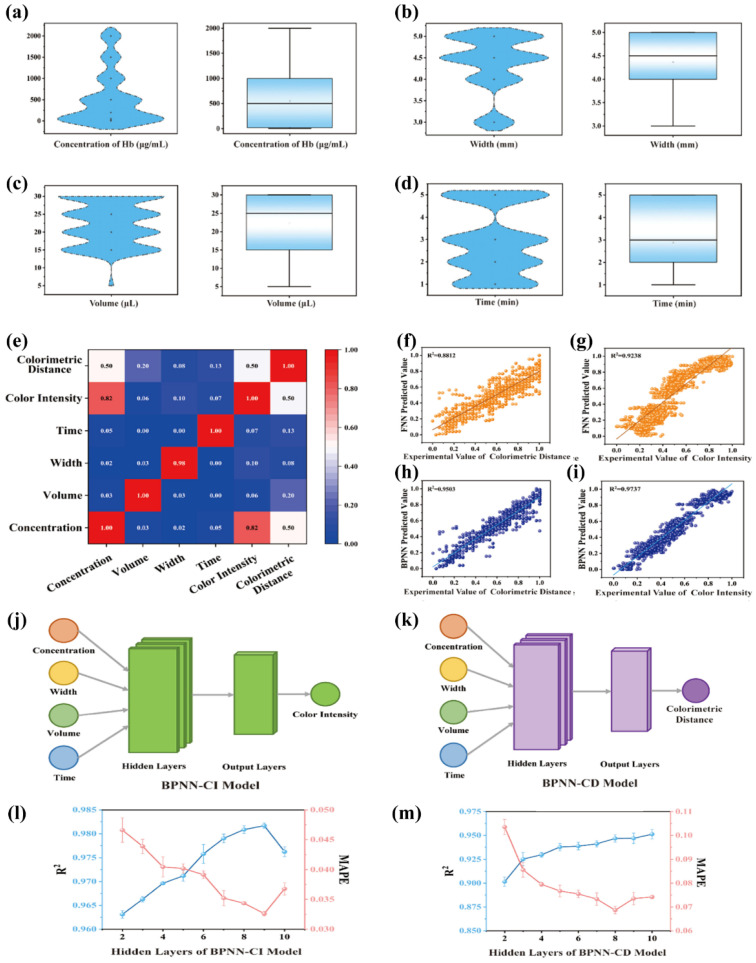
Establishment of the CNGCOS Model and Data Analysis. Statistical distribution of the four controllable variables, including (**a**) concentration, (**b**) width, (**c**) volume, and (**d**) time, in the cyclic optimization strategy. (**e**) Maximum information coefficient (MIC) correlation diagram. Data fitting results were based on feedforward neural network (FNN) with (**f**) CD and (**g**) CI as optimization targets. Data fitting results based on backpropagation neural network (BPNN) with (**h**) CD and (**i**) CI as optimization targets. Schematic diagram of the network structure for (**j**) BPNN-CI and (**k**) BPNN-CD. Optimization of the number of hidden layers in (**l**) BPNN-CI and (**m**) BPNN-CD. Adapted with permission from Ref. [95]. Copyright 2025, Kangzheng Lv et al.

The distributions of the input variables were presented as violin plots and box plots, showing that Hb concentration, width, volume, and time were all designed to vary over broad ranges (Figure 6a–d). Maximal information coefficient analysis showed that concentration and volume were strongly related to the output signals, whereas the other variables were also not independent of the outputs (Figure 6e). In practice, the backpropagation neural network showed better fitting performance than the feedforward neural network for both colorimetric distance and color intensity (Figure 6f–i). The architectures of the BPNN-CI and BPNN-CD models were also presented separately (Figure 6j,k) [133], and the changes in R^2^ and MAPE according to the number of hidden layers showed that an appropriate network depth is important for prediction accuracy (Figure 6l,m). These results suggest that μPAD performance is determined not by the independent effect of each variable, but by nonlinear interactions among multiple variables [134,135]. These findings also show that one-factor-at-a-time optimization of channel structure or operating conditions is not sufficient to explain the actual mechanism of signal generation. Because the effect of a given variable can change depending on the conditions of other variables, μPAD design and operation need to be addressed by considering multivariable interactions rather than by adjusting single variables alone. From this perspective, the BPNN-based model functions not simply as a prediction tool but as a core framework that quantitatively identifies which combinations of input variables govern the colorimetric response and links this information to the subsequent optimization step [136,137]. In practical terms, this approach can reduce trial-and-error because the trained model predicts the output response for candidate parameter combinations before additional experiments are performed, while the genetic algorithm guides the search toward promising regions of the design space instead of relying on exhaustive or sequential manual testing.

**Figure 7:**
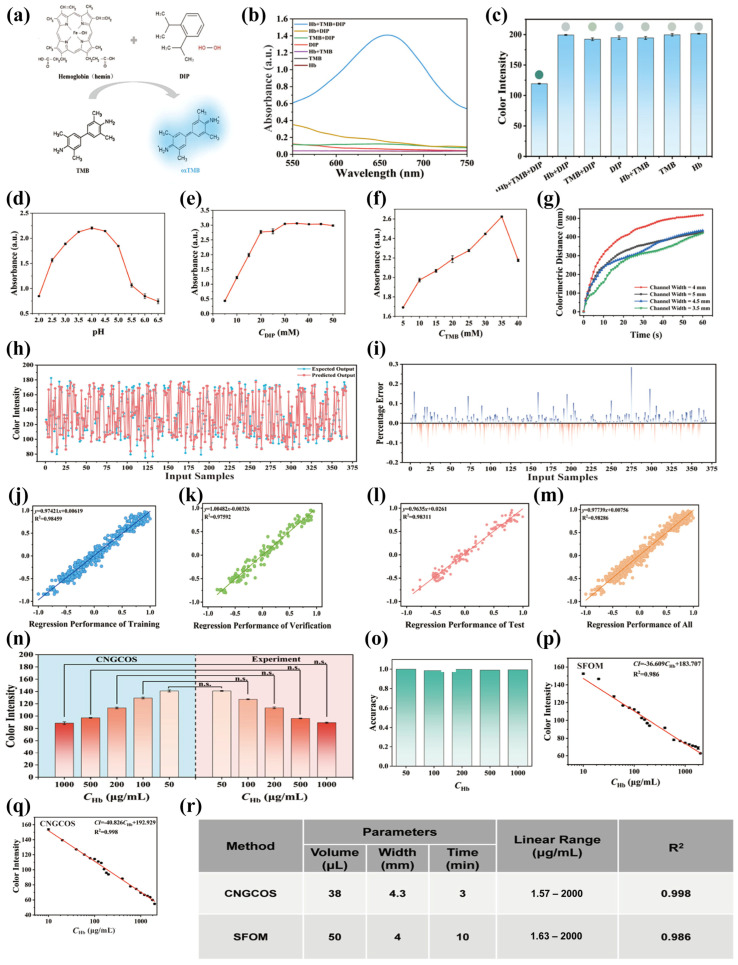
CNGCOS-assisted μPADs for the detection of hemoglobin (Hb) with CI as output signal. (**a**) Principle of Hb detection. (**b**) UV–vis absorbance spectra and (**c**) CI obtained for the detection of Hb. Optimization of the preparation parameters, including (**d**) pH value, (**e**) 3,5-diisopropylbenzene hydroperoxide (DIP) concentration, (**f**) 3,3′,5,5′-tetramethylbenzidine (TMB) concentration, and (**g**) width of paper by employing traditional single-factor optimization method (SFOM). (**h**) Fitting of predicted CI values versus actual values from BPNN output. (**i**) Percentage error between predicted CI values and actual values from the BPNN output. Fitting regression of (**j**) training set, (**k**) validation set, (**l**) test set, and (**m**) overall data set by BPNN-CI. (**n**) Predicted and experimental values and (**o**) accuracies for different concentrations of Hb. Standard curves obtained for (**p**) SFOM-assisted μPADs and (**q**) CNGCOS-assisted μPADs. (**r**) Data comparison between the two methods. Adapted with permission from Ref. [95]. Copyright 2025, Kangzheng Lv et al.

CNGCOS was then applied to actual μPAD optimization. In the colorimetric detection system based on the peroxidase-like activity of Hb, the reaction scheme and absorbance spectra of hemin, TMB, and DIP illustrate the color-generation mechanism of the system (Figure 7a,b), and the effects of reaction conditions, including color intensity, pH, DIP concentration, TMB concentration, channel width, and reaction time, show that the output signal depends sensitively on multivariable conditions (Figure 7c–g). These results also clarify why SFOM is limited for this type of μPAD design. In SFOM, pH, reagent concentration, paper width, and reaction time are optimized one by one, which makes the optimization process easier to interpret but less efficient when the variables interact. By contrast, CNGCOS uses the learned relationship between multiple inputs and output signals to evaluate parameter combinations more systematically. Comparison between predicted and expected values, together with percentage error analysis, confirmed that the proposed model maintained stable predictive performance overall (Figure 7h,i), and the regression results for the training, verification, test, and full datasets also showed strong correlations (Figure 7j–m). Based on these results, direct comparison between CNGCOS and the experiment showed that the optimized conditions produced a stronger color intensity response (Figure 7n), while accuracy across concentrations remained stable (Figure 7o). In particular, the calibration and parameter comparison between CNGCOS and SFOM showed that the proposed strategy provided higher R^2^, shorter reaction time, smaller sample volume, and a wider effective operating range (Figure 7p–r). These improvements indicate that the ML-driven strategy did not merely reproduce the results of conventional optimization but improved experimental efficiency by identifying favorable conditions with less reliance on repeated single-variable screening. In this case, the role of AI extends beyond assisting signal interpretation and can be understood as a core design framework that iteratively updates the fabrication and operating conditions of the μPAD to derive a better device configuration [138,139]. These advantages were maintained in real clinical samples.

**Figure 8:**
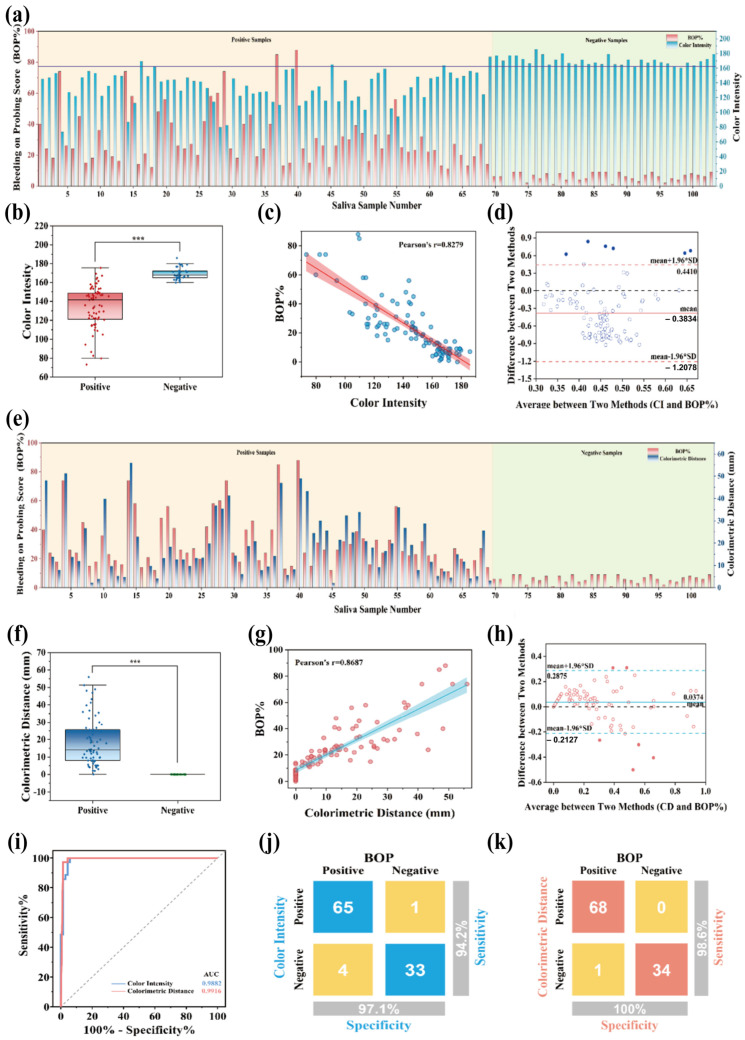
Clinical application of CNGCOS-assisted μPADs for periodontitis early detection in saliva samples. (**a**) CI and BOP% detection values of Hb in saliva samples. (**b**) Box plot of CI of positive samples (n = 69) versus negative samples (n = 34). (**c**) Correlation analysis between the CI and BOP%. (**d**) Bland–Altman analysis between CI and BOP%. (**e**) CD and BOP% detection values of Hb in saliva samples. (**f**) Box plot of CD of positive samples (n = 69) versus negative samples (n = 34). (**g**) Correlation analysis between CD and BOP%. (**h**) Bland–Altman analysis between CD and BOP%. (**i**) ROC curve analysis obtained from clinical saliva samples by the CNGCOS. AUC: area under the curve. Evaluation of the sensitivity and specificity of (**j**) CI and (**k**) CD compared with BOP% using a confusion matrix (*** *p* ≤ 0.001). Adapted with permission from Ref. [95]. Copyright 2025, Kangzheng Lv et al.

The authors applied CNGCOS-assisted μPADs to 103 clinical saliva samples. In the color intensity-based analysis, the overall distribution relative to BOP% was presented (Figure 8a), and significant differences between the positive and negative groups, together with strong correlations, were confirmed through box plots and correlation analysis (Figure 8b,c). Bland–Altman analysis showed the agreement between the two methods (Figure 8d), and the same type of analysis was repeated for colorimetric distance, further validating its relationship with BOP% (Figure 8e–h). The ROC curve showed high diagnostic performance for both color intensity and colorimetric distance (Figure 8i), and the confusion matrix indicated high sensitivity and specificity for both readouts (Figure 8j,k). These results show that the variable combinations obtained during the optimization stage are not only valid under laboratory conditions but can also be maintained in complex biological matrices such as real saliva samples. The fact that both color intensity and colorimetric distance showed consistent diagnostic performance also suggests that the proposed framework does not rely excessively on a single signal but instead provides a relatively robust readout structure [129,140]. In this case, ML moves beyond a supporting tool for correcting μPAD signals and functions as a core optimization framework that links device parameter optimization with actual clinical application. These findings further suggest that AI-based optimization can shift μPAD design from experimental exploration toward a data-driven and reproducible design process [141,142].

### 3.3. Optical Biosensor Design: AI-Based Surrogate Prediction of Sensor Parameters

In optical biosensors, structural parameters and optical responses directly govern resonance behavior and sensitivity, so sensor design needs to be understood as a parameter prediction problem involving multiple continuous variables [143,144]. Focusing on this point, Ahmed et al. proposed an ML-based approach in which core radius, cladding radius, pitch, analyte, and wavelength were used as input variables to predict key optical parameters, including effective refractive index, power profile, effective area, confinement loss, and sensitivity [97].

**Figure 9:**
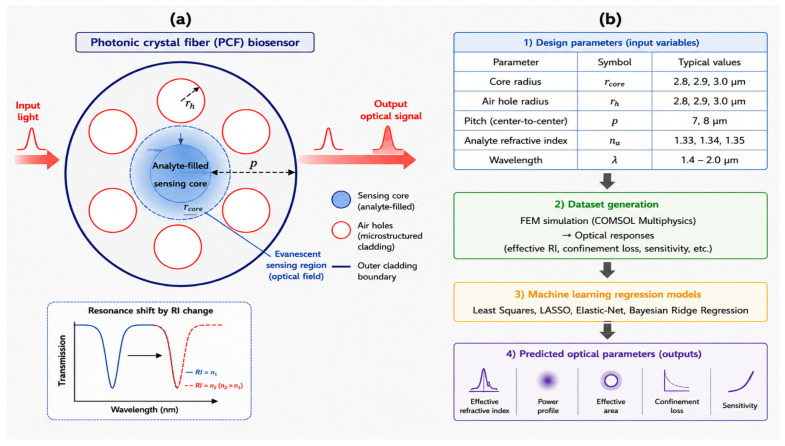
ML-assisted optical biosensor design framework. (**a**) Cross-sectional structure of the proposed photonic crystal fiber biosensor. (**b**) Design-dependent input variables and predicted optical output parameters. Adapted with permission from Ref. [97]. Copyright 2023, Ahmed et al.

Figure 9 shows a machine-learning-assisted PCF biosensor design framework. The sensor structure consists of an analyte-filled sensing core surrounded by air holes, where refractive index changes in the analyte region induce a resonance shift in the output optical signal (Figure 9a). Structural and optical parameters such as core radius, air-hole radius, pitch, analyte refractive index, and wavelength are used as input variables for FEM-based dataset generation, followed by regression model training to predict optical parameters including effective refractive index, confinement loss, effective area, and sensitivity (Figure 9b). This shows that optical biosensor design can move beyond repeated numerical simulation and be reformulated as a learnable problem that captures the relationships between key design variables and performance metrics. Based on a dataset generated by COMSOL simulations, the authors compared least squares, LASSO, Elastic-Net, and Bayesian ridge regression, and showed particularly strong agreement between predicted and simulated values for effective refractive index prediction.

**Figure 10:**
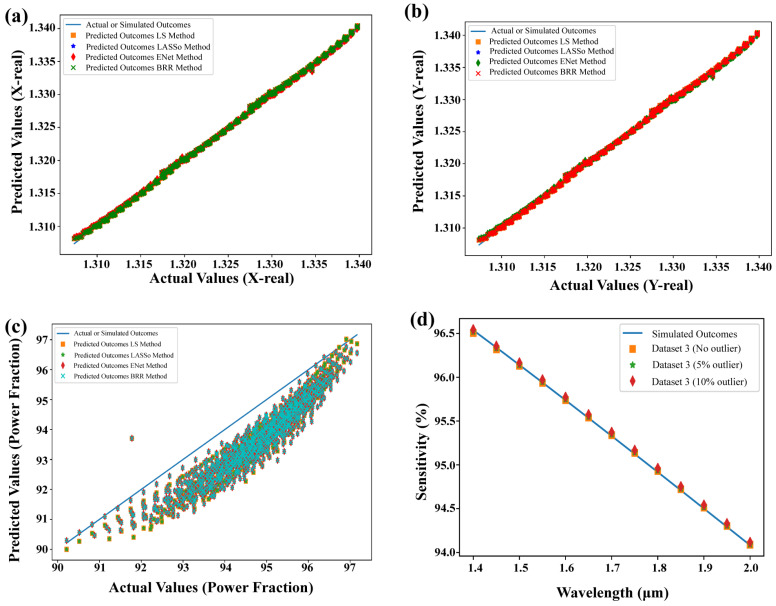
Representative data-based summary of ML-assisted optical biosensor prediction and robustness evaluation. (**a**) Actual-versus-predicted comparison for effective refractive index prediction in the X-real direction. (**b**) Actual-versus-predicted comparison for effective refractive index prediction in the Y-real direction. (**c**) Actual-versus-predicted comparison for core power fraction prediction. (**d**) Representative sensitivity prediction under no-outlier, 5% outlier, and 10% outlier conditions. The integrated figure summarizes representative prediction accuracy and robustness results while avoiding repetitive data-only plots. Adapted with permission from Ref. [97]. Copyright 2023, Ahmed et al.

The effective refractive index was first evaluated as a representative optical descriptor for ML-assisted biosensor prediction. In the X-real direction, the direct comparison between actual and predicted values showed high agreement across all models, with most data points closely distributed along the diagonal line (Figure 10a). The same trend was observed in the Y-real direction, where the errors in the actual–predicted comparison remained very small (Figure 10b). In fact, for the real part of the effective refractive index, most models showed an R^2^ above 0.99, with least squares and Bayesian ridge regression giving the closest agreement. These results indicate that the key physical parameters of optical biosensors can be approximated without repeated simulations when sufficient training data are available [145,146]. A key point of this study is that the prediction does not stop at a single optical parameter but extends to actual sensing performance metrics. Based on the predicted effective index and power fraction, performance metrics such as sensitivity, confinement loss, and core power fraction were calculated. In the comparison between actual and predicted values, most data points were distributed close to the diagonal, demonstrating the reliability of the power fraction prediction (Figure 10c). These results suggest that ML functions not simply as a regression tool for rapidly fitting design values, but as a surrogate prediction framework that approximates the broader set of physical quantities governing optical biosensor performance. The study also examined model stability in relation to data quality. As the amount of training data increased, overall model performance improved, and sensitivity prediction remained relatively stable even when 5–10% outliers were included. In the representative sensitivity analysis, the predicted sensitivity showed trends close to the simulated outcome under the no-outlier, 5% outlier, and 10% outlier conditions (Figure 10d). These results suggest that ML-based optical biosensor design is not valid only under ideal simulation conditions but can also function as a practical predictive tool under a certain level of data variability. They further show that the relationship between structural parameters and optical responses is not simply fitted to a specific dataset but can remain relatively stable even in the presence of a moderate level of noise and outliers [147,148]. ML models can therefore be used not only to replace repeated simulation results, but also to rapidly screen diverse conditions and identify promising structural candidates at the early stage of design [149,150]. The significance of this study thus lies in showing that AI does not directly replace structural design with optical biosensors but instead functions as a key predictive tool that complements or reduces repeated numerical simulations while accelerating the exploration of feasible design conditions.

Taken together, these case studies show that AI plays different roles depending on the sensor platform, including complex signal interpretation, optimization of fabrication conditions, and prediction of key parameters. This trend indicates that AI is moving beyond a simple post-processing tool and is becoming a central means of design and optimization in biosensor fabrication and parameter optimization (Table 4).

## 4. Conclusions and Outlooks

The adoption of AI is shifting biosensor research away from the conventional approach in which the discovery of molecular recognition elements, sensor fabrication, and signal interpretation were treated as separate stages [154,155]. At the biorecognition stage, in silico aptamer design, smart-SELEX, and bio-inspired peptide design have enabled the more systematic identification of target-binding candidates, while at the stages of sensor fabrication and signal interpretation, the scope of AI application has expanded to include the interpretation of complex electrochemical signals, cyclic optimization of μPADs, and prediction of optical biosensor parameters [68,69,70,92,95,97]. These trends show that AI is moving beyond a simple data-processing tool and is developing into an approach that improves design and optimization across the overall biosensor development process.

These advances have made candidate discovery and the exploration of design variables more systematic and have enabled hidden patterns in complex signals, as well as relationships among variables, to be identified quantitatively. At the same time, they have shown the potential to improve both design efficiency and analytical performance while reducing dependence on repeated experiments and numerical simulations [156,157,158]. Nevertheless, several challenges remain before AI-assisted biosensors can be translated into broadly reliable platforms. Many reported models are still trained using small experimental datasets or idealized simulation data, which can increase the risk of overfitting and limit generalizability [159,160]. Therefore, future studies should place greater emphasis on larger and more diverse datasets, independent test sets, external validation using real biological or environmental samples, and robustness evaluation across different sensor batches, operators, and measurement conditions. In addition, biosensor responses can be affected by nonspecific binding, matrix effects, fabrication variability, storage conditions, and environmental changes [161,162]; thus, AI models should be assessed under practical stress-test conditions rather than only under optimized laboratory settings.

Another important issue is model interpretability. Although tools such as SHAP analysis have begun to be used to identify important input variables, explainable AI should be more actively incorporated into clinical biosensor development. Interpretable models can help clarify whether model predictions are based on physically and biologically meaningful features rather than dataset-specific artifacts, thereby reducing the risk of misleading diagnostic outputs [163,164]. The integration of explainable AI, uncertainty estimation, and mechanism-informed modeling would therefore be valuable for connecting prediction results with the underlying physicochemical and biological processes of biosensor responses.

Practical challenges also remain in AI-guided fabrication. The construction of reliable fabrication datasets requires systematic experimental design, consistent reporting of processing parameters, and sufficient replication to capture batch-to-batch variability. Furthermore, optimization results obtained in one laboratory may not be directly transferable to other laboratories because of differences in materials, instruments, environmental conditions, and fabrication protocols. For AI-guided fabrication to become scalable, future studies should consider standardized datasets, reproducible experimental workflows, cross-laboratory validation, and manufacturing-compatible optimization strategies.

Future AI-assisted biosensor development should therefore move toward integrated and closed-loop workflows rather than isolated applications of individual algorithms. In such workflows, molecular design results can guide sensor fabrication, fabrication data can update optimization models, and signal-analysis outcomes can feed back into the next design cycle. Ultimately, the significance of AI-based biosensor research lies not simply in automating individual steps, but in establishing a basis for advancing biosensors into more precise, reproducible, interpretable, and adaptive analytical platforms by linking bioprobe design, fabrication optimization, and signal interpretation into a single data-driven development workflow.

## Figures and Tables

**Table 1:** Representative artificial intelligence-assisted bioprobe engineering strategies for biosensor development.

Target	Bioprobe	AI-Assisted Task	Biosensor Readout	[Ref]
β-parvalbumin	DNA aptamer	Sequence design and screening	Square wave voltammetry	[68]
Severe acute respiratory syndrome coronavirus 2 (SARS-CoV-2)	Peptide receptor	Peptide design and signal analysis	Cyclic voltammetry	[69]
Ammonium ion (NH4+)	RNA aptamer	Binding prediction and ranking	Electrochemical impedance spectroscopy	[70]
Okadaic acid	DNA aptamer	In silico optimization	Square wave voltammetry	[71]
Target-specific analyte	DNA aptamer	Truncation and docking	Electrochemical	[72]
*E. coli* O157:H7	Peptide receptor	Bioinformatic peptide design	Electrochemical	[73]

**Table 2:** Single Feature Analysis Using Random Forest Regression.

Feature	C_Co,1_	C′_Cu_	C_Cu_	A_Cu_	C_Co,1_/C′_Cu_	C_Co,1_/C_Cu_	C_Co,1_/A_Cu_	C′_Cu_/C_Cu_	C′_Cu_/A_Cu_	C_Cu_/A_Cu_	C_Co,1_
R^2^_testing_	0.860	0.947	0.846	0.875	0.970	0.805	0.893	0.978	0.964	0.860	0.860
RMSE_training_	0.461	0.282	0.482	0.435	0.209	0.542	0.398	0.180	0.231	0.456	0.461
R^2^_testing_	0.587	0.664	0.317	0.399	0.797	0.001	0.296	0.841	0.745	0.127	0.587
RMSE_testing_	0.812	0.732	1.114	1.002	0.572	1.522	1.096	0.499	0.635	1.244	0.812

**Table 3:** Machine Learning Model Comparison.

Models	LR	SVM	kNN	DT	RF	ET	GB	XGB
R^2^_testing_	0.940	0.904	0.876	1.000	0.989	1.000	1.000	1.000
RMSE_training_	0.304	0.386	0.439	0.000	0.132	0.000	0.009	0.001
R^2^_testing_	0.902	0.874	0.798	0.837	0.916	0.915	0.889	0.904
RMSE_testing_	0.390	0.442	0.561	0.353	0.412	0.361	0.513	0.386

**Table 4:** Input variables, ML models, and output targets in AI-assisted sensor fabrication and optimization studies.

Sensor Platform	Target/Application	Input Variables	Machine Learning Models	Output/Prediction Target	[Ref]
Dual-metal electrochemical sensor (Co@Cu/GC)	Creatinine detection	Peak height, peak ratio, multi-peak voltammetric features	Random Forest, Linear Regression, SVM, kNN, Decision Tree, Extra Trees, Gradient Boosting, XGBoost	Creatinine concentration	[92]
Paper-based microfluidic analytical device (μPAD)	Hemoglobin-based colorimetric sensing/periodontitis-related diagnosis	Hb concentration, test strip width, sample volume, reaction time	Computer vision, Backpropagation Neural Network (BPNN), Genetic Algorithm (GA)	CI, CD, and optimal operating conditions	[95]
Optical biosensor	Optical parameter prediction/design acceleration	Core radius, cladding radius, pitch, analyte, wavelength	Least Squares, LASSO, Elastic-Net, Bayesian Ridge Regression	Key optical parameters and sensitivity	[97]
Electrochemical glucose sensor	Low-concentration glucose detection	Electrochemical curve features and experimental conditions	MLR, DT, ANN, RF, XGBoost	Glucose concentration and optimal experimental conditions	[151]
Wearable microfluidic colorimetric sensor	Tear biomarker monitoring	RGB color data under varying pH and color temperature conditions	1D-CNN-GRU, 3D-CNN-GRU	Concentrations of pH, vitamin C, Ca^2+^, and proteins	[152]
Graphene-gold hybrid metasurface biosensor	Isoquercitrin biomarker detection	Spectral features, geometric parameters, graphene chemical potential	RF, SVM, Neural Network ensemble	Isoquercitrin concentration/spectral-response prediction	[153]

## Data Availability

No new data were created or analyzed in this study. Data sharing is not applicable to this article.
